# AT2 activation does not influence brain damage in the early phase after experimental traumatic brain injury in male mice

**DOI:** 10.1038/s41598-022-18338-x

**Published:** 2022-08-22

**Authors:** Ralph Timaru-Kast, Andreas Garcia Bardon, Clara Luh, Shila P. Coronel-Castello, Phuriphong Songarj, Eva-Verena Griemert, Tobias J. Krämer, Anne Sebastiani, Ulrike Muscha Steckelings, Serge C. Thal

**Affiliations:** 1 Department of Anesthesiology, University Medical Center of the Johannes Gutenberg University, Langenbeckstrasse 1, 55131 Mainz, Germany; 2 Focus Program Translational Neuroscience, University Medical Center of the Johannes Gutenberg University, Mainz, Germany; 3 Department of Anesthesiology, Faculty of Medicine, Siriraj Hospital, Mahidol University, 2 Prannok Road Bangkoknoi, Bangkok, 10700 Thailand; 4 Faculty of Health, University of Witten/Herdecke, Witten, Germany; 5 Department of Anesthesiology, HELIOS University Hospital Wuppertal University of Witten/Herdecke, Heusnerstrasse 40, 42283 Wuppertal, Germany; 6 Department of Cardiovascular and Renal Research, Institute of Molecular Medicine, University of Southern Denmark, Odense, Denmark

**Keywords:** Brain injuries, Experimental models of disease, Preclinical research, Trauma, Post-traumatic stress disorder, Cell death in the nervous system, Regeneration and repair in the nervous system

## Abstract

Antagonism of the angiotensin II type 1 receptor (AT1) improves neurological function and reduces brain damage after experimental traumatic brain injury (TBI), which may be partly a result of enhanced indirect angiotensin II type 2 receptor (AT2) stimulation. AT2 stimulation was demonstrated to be neuroprotective via anti-inflammatory, vasodilatory, and neuroregenerative mechanisms in experimental cerebral pathology models. We recently demonstrated an upregulation of AT2 after TBI suggesting a protective mechanism. The present study investigated the effect of post-traumatic (5 days after TBI) AT2 activation via high and low doses of a selective AT2 agonist, compound 21 (C21), compared to vehicle-treated controls. No differences in the extent of the TBI-induced lesions were found between both doses of C21 and the controls. We then tested AT2-knockdown animals for secondary brain damage after experimental TBI. Lesion volume and neurological outcomes in AT2-deficient mice were similar to those in wild-type control mice at both 24 h and 5 days post-trauma. Thus, in contrast to AT1 antagonism, AT2 modulation does not influence the initial pathophysiological mechanisms of TBI in the first 5 days after the insult, indicating that AT2 plays only a minor role in the early phase following trauma-induced brain damage.

## Introduction

Traumatic brain injury (TBI) is the most common cause of trauma-related death and disability in industrialized countries^1^. Following the primary injury, mechanisms such as cerebral inflammation lead to further (secondary) brain damage^2^. Increasing evidence indicates that the intrinsic central renin-angiotensin system (RAS) is involved in the pathophysiology of various cerebral pathologies, including TBI^3^. The RAS effector peptide angiotensin II (AngII) binds predominately to two G protein-coupled transmembrane receptors known as angiotensin II receptor type 1 (AT1) and type 2 (AT2). RAS-mediated vasoconstriction and inflammation are produced by AngII binding to AT1^4^. Recent studies have revealed that blockade of AT1 improved neurological outcomes and reduced secondary brain damage after experimental TBI ^5–7^. Our previous data indicate that there is also a post-traumatic upregulation of AT2 in the first days after TBI in the perilesional tissue ^5^, which may be related to the initiation of protective repair mechanisms^8^. Indeed, RAS signaling via AT2 is known to mediate its anti-inflammatory and neuro-regenerative functions^9^. Upregulated in various cerebral insults^10–16^, AT2 activation may counterbalance pathological processes mediated by AT1^17,18^. Because reducing AT1 activity augments AT2 activation ^19^, the neuroprotective properties of AT1 antagonists may actually be related to enhancement of AT2 function. Iwai et al. reported increased infarct size and neurological impairment in AT2-knockdown mice (AT2^−/y^) after permanent middle cerebral artery occlusion (MCAO)^20^. Furthermore, in AT2^−/y^ and under concomitant AT2 antagonism, AT1 blockade was less effective in reducing the infarct size and improving neurological outcomes, suggesting that indirect AT2 stimulation underlies AT1 inhibition-mediated protection^17^. Signal transduction after AT2 involves the neurotrophic tyrosine kinase receptor type 1 (NTRK1) and the extracellular signal-regulated kinases 1 and 2, as well as protein kinase B and brain-derived neurotrophic factor (BDNF)^10,21–23^.

Long-term treatment of mice with an AT2 agonist peptide was shown to improve anatomical and functional markers of recovery after closed-head TBI^24^, and post-MCAO treatment of mice with a non-peptide specific and selective AT2 agonist, compound 21 (C21), was found by Schwengel et al. to reduce mortality and neurological impairment^25^, summarized in^26^. These results suggest that AT2 activation may be a novel approach to treating TBI. In the present study, we aimed to clarify the significance of AT2 activation in the context of TBI.

To this end, we investigated the role of AT2 in secondary brain damage, cerebral inflammation, and neurologic outcomes after TBI in mice by (1) treating mice with high or low doses of C21 and comparing the results to those in vehicle-treated mice; and (2) analyzing the same outcomes after TBI in AT2-knockdown versus wild-type mice. We also analyzed gene expression of cytokines and neurotrophins to elucidate the putative underlying molecular mechanisms involved.

## Materials and methods

### Animals

In the first (pharmacology) study, C57Bl6N adult male mice (Charles River Laboratory, Sulzfeld, Germany) were used; in the second (AT2 knockdown) study, AT2^−/y^ (Agtr2^tm1a(EUCOMM)Wtsi^) and wild-type litter mates (WT; AT2^+/y^; C57Bl6N background) were investigated. The studies were performed with the approval of the Animal Care and Ethics Committee of Rhineland-Palatinate, Germany in accordance with the institutional guidelines of the Johannes Gutenberg University, Mainz (protocol numbers: 23177–07/G07-1–021 and 23,177–07/G13-1–046) and in compliance with the ARRIVE guidelines. The animals were kept under controlled light and environmental conditions (12-h dark/light cycle, 23 ± 1 °C, 55% ± 5% relative humidity), and had free access to food (Altromin, Germany) and water at all times before and after the experiments.

### Experimental traumatic brain injury

The animals were anesthetized with sevoflurane (4% by volume) and an air mixture (40% O_2_ and 60% N_2_) via face mask. The rectal temperature was maintained constant at 37 °C by a feedback-controlled heating pad (Hugo Sachs, March-Hugstetten, Germany). Experimental brain trauma was induced by controlled cortical impact (CCI) as described previously ^27^. Briefly, the animal’s head was fixed in a stereotactic frame (Kopf Instruments, Tujunga, LA, USA) and a large craniotomy (4 × 4 mm^2^) was performed above the right parietal cortex between the sagittal, lambdoid, and coronal sutures and the insertion of the temporal muscle. A custom-fabricated pneumatic impactor (L. Kopacz, Mainz, Germany) was placed perpendicularly to the brain surface and the impactor tip (diameter, 3 mm) centered in the middle of the craniotomy. The impact parameters were as follows: velocity, 7.5 m/s; duration, 200 ms; brain penetration, 1.5 (study I) and 1.0 mm (study I confirmatory study and study II). Immediately after CCI, the craniotomy was closed with conventional tissue glue (Histoacryl; Braun-Melsungen, Melsungen, Germany) and filament sutures. The animals were randomly assigned to groups, which were followed-up for 15 min (primary injury), 24 h (1 day post injury, 1dpi), or 120 h post-CCI (5 days post injury, 5dpi). Animals in the 15 min group remained anesthetized on the heating pad until their brains were removed; animals in the other groups were placed in their individual cages at the end of surgery and allowed to recover for 6 h in an incubator heated to 33 °C and with 35% humidity (IC8000; Dräger, Germany).

During surgery, the blood pressure was measured 5 min before and after CCI at the tail using a modified NIBP system (RTBP 2000; Kent, USA) as previously described^28^. Additionally, the blood pressure was determined daily for 8 days before (training phase) and 4 days after CCI. Cuff pressure signals were recorded with a sample rate of 100 Hz (A/D converter: PCI 9112; Adlink Technology, Taiwan; PC software: Dasylab 5.0; measX, Germany) and analyzed offline (Flexpro 6.0, Weisang, Germany) for systolic blood pressure. Perioperative body temperature was measured by a rectal temperature probe (Physitemp; Clifton, NJ, USA).

### Experimental groups

#### Study I: Effect of a 5-day post-CCI treatment with the selective AT2 agonist C21

WT mice were randomized (*n* = 9/group) to daily intraperitoneal (i.p.) injection with low- (0.03 mg/kg; LD) or high- (0.1 mg/kg; HD) dose C21 or vehicle (saline 0.9%). The injections started 30 min after TBI and were repeated every 24 h until day 4. The cortical cytokine and neurotrophin gene expressions of the CCI groups were compared to those of naïve (non-operated) animals (n = 4). In order to differentiate primary from secondary injury, a group of WT mice (*n* = 8) was anesthetized, underwent CCI, and then sacrificed 15 min post-TBI, brains were then removed and analyzed for primary injury volume. The protocol of study I was then repeated in a confirmatory study using modified CCI device settings (see above) with eight animals/group.

#### Study II: TBI in AT2-knockdown mice compared to WT mice

AT2^−/y^ and AT2^+/y^ mice were followed up for either 24 h (n = 12/group) or 5 days after TBI (n = 6/group). Neurological assessment was performed before and after TBI. At the end of the follow-up period, the brains were removed and examined immunohistologically for cerebral lesion volume and activated microglia/macrophages, and cytokine and neurotrophin expressions were quantified via quantitative real-time polymerase chain reaction (qPCR). To differentiate primary from secondary injury, one group of WT mice (*n* = 6) and one group of AT2^-/y^ mice (*n* = 5) was anesthetized, underwent CCI, and then sacrificed 15 min post-TBI, at which point the brains were removed and analyzed for primary injury volume.

### Neurological assessment

The neurological outcome was determined by neurological severity score (NSS^29^, in study I), by modified NSS (mNSS^7^, in study II), and body weight 1 day before and 24, 72, and 120 h after CCI by an investigator blinded toward group allocation.

In study I, to calculate NSS, general behavior, alertness, motor ability, and balance were rated over 10 different tasks. Each task was scored from 0 (normal) to 1 (failed task). One day prior to their surgeries, all animals were tested with the NSS. Healthy animals were required to pass the test with 0 or 1 point to be enrolled in the study.

In study II different and older mice were used. Therefore, a modified NSS (mNSS) was applied to have a more specific differentiation of neurological status^7^. The neurological outcome was determined by mNSS, modified after Tsenter et al. (2008)^29^, 1 day before and 24, 72, and 120 h after CCI by an investigator blind toward the group allocation. To calculate mNSS^7^, general behavior, alertness, motor ability and balance were rated with 10 different tasks. Each task was scored from 0 (normal) up to 3 (failed task). The mNSS ranges from 0 (healthy) to 16 (severely impaired) points^7,29,30^ (Table 1).

**Table 1:** Modified Neurological Severity Score.

	Points	
**1. Exit from circle**	< 30 s	0
	For 30 s	1
	For 60 s	2
	> 2 min	3
**2. Startle reflex**	Present	0
	Absent	1
**3. General behavioral deficit**		
Seeking behavior	Present	0
	Absent	1
Walk straight	Present	0
	Absent	1
**4. Coordination** (Criteria: 0P: no impairment; 1P: feet misplacement, unstable; 2P: stops moving)		
Beam walking 3 cm	Score	(0–2)
Beam walking 1,5 cm	Score	(0–2)
Beam walking 1 cm	Score	(0–2)
**5. Balance** (Criteria: 0P: grips stick with 4 paws; 1P: 1–4 paws not gripping)		
Round stick Square stick	Score	(0–1)
	Score	(0–1)
**6. Motor deficit**		
Hemiparesis	Absent	0
	One Foot	1
	Present	2

The modified Neurological Severity Score was designed on the basis of the Neurological Severity Score introduced by Tsenter and colleagues ^29^. The mNSS focusses on motoric function and behavioral deficits and was performed 1 day before CCI and on posttraumatic day 1, 3, and 5 after experimental TBI.

### Histologic evaluation of secondary brain damage and immunohistochemistry

For tissue evaluation, the brains were frozen in powdered dry ice after dissection and stored at − 20 °C. They were then cut in the coronal plane with a cryostat (HM 560 Cryo-Star; Thermo Fisher Scientific, Germany). The first slide was defined according to the first section corresponding to bregma + 3.14 mm in the Mouse Brain Library (www.mbl.org). 16 Sects. (10 µm) were collected at 500 µm intervals and placed on Superfrost +™ slides (Thermo Fisher Scientific). In cresyl violet stained sections, total area of both hemispheres and the injured brain tissue area were determined for each section and animal using a computerized image analysis system (Delta Pix Insight; Maalov, Denmark) by an investigator blinded to group allocations. The total hemispheric brain volumes and the lesion volumes were calculated by following formula: 0.5 [mm] × (area of slide 1 [mm^2^] + area of slide 2 [mm^2^] + … + area of slide 16 [mm^2^])^5^. For immunohistochemical staining of activated microglia/macrophages, cryosections were fixed in paraformaldehyde in phosphate buffered saline, incubated with blocking serum, and then incubated overnight at room temperature with anti-ionized calcium-binding adapter molecule-1 (rabbit anti mouse, anti-Iba-1 antibody; Wako Chemicals, Neuss, Germany, catalogue number: #019–19,741). The sections were washed, incubated with secondary biotin-conjugated antibodies (goat anti-rabbit IgG; Merck; Darmstadt, Germany) and processed according to the manufacturer’s instructions using a Vectastain Elite ABC Kit (Vector Laboratories, Burlingame, CA, USA). Images were taken at 20 × magnification (Axiovert, Zeiss, Germany). The total number of positive cells ( +) was counted at bregma − 1.28 mm by two investigators blinded to randomization using ImageJ (National Institutes of Health, USA). Iba1-immunolabeled cells with appropriate morphology and appearance^31^ were identified as activated microglia/macrophages and assessed in a region of interest (ROI) of 0.52 × 0.65 mm^2^ in the cortical tissue adjacent to the lesion.

### Gene expression analysis

Brain tissue samples were excised during histological cryosectioning. Samples from lesion and perilesional tissue (right upper quadrant) and corresponding contralateral cortical tissue (left upper quadrant) were collected, snap frozen in liquid nitrogen, and stored at − 80 °C. qPCR was performed as previously described^7,32^. Using specific primers^7,33,34^ (Table 2), absolute copy numbers of BDNF, growth associated protein 43 (GAP43), NTRK1, NTRK2, transforming growth factor beta (TGFβ), interleukin 1β (IL1β), and IL6 were calculated, and were then normalized against the absolute copy numbers of cyclophilin A (PPIA)^34^.

**Table 2:** Primers for quantitative real-time PCR.

PCR assay (amplicon size)	Oligonucleotide Sequence (5′–3′)	GenBank No
PPIA (146 bp)	F: 5′-GCGTCTSCTTCGAGCTGTT-3′	NM_008907
	R: 5′-RAAGTCACCACCCTGGCA-3′	
BDNF (155 bp)	F: 5′-ACTTGGCCTACCCAGGTG-3′	NM_001048139
	R: 5′-GTTGGGCCGAACCTTCT-3′	NM_001048142
GAP43 (150 bp)	F: 5′-AGGAGCCTAAACAAGCCGAT-3′	NM_008083
	R: 5′-CGTCTACAGCGTCTTTCTCCTC-3′	
NTRK1 (99 bp)	F: 5′-GCCTAACCATCGTGAAGAGTG-3′	NM_001033124
	R: 5′-CCAACGCATTGGAGGACAGAT-3′	
NTRK2 (100 bp)	F: 5′-CTGGGGCTTATGCCTGCTG-3′	NM_008745
	R: 5′-AGGCTCAGTACACCAAATCCTA-3′	
TGFβ (142 bp)	F: 5′-CTTCAATACGTCAGACATTCGGG-3′	NM_011577
	R: 5′-GTAACGCCAGGAATTGTTGCTA-3′	
IL1β (348 bp)	F: 5′-GTGCTGTCGGACCCATATGAG-3′	NM_008361
	R: 5′-CAGGAAGACAGGCTTGTGCTC-3′	
IL6 (471 bp)	F: 5′-TCGTGGAAATGAGAAAAGAGTTG-3′	NM_031168
	R: 5′-TATGCTTAGGCATAACGCACTAG-3′	

Mouse specific primers and probes for qPCR; *F* forward, *R* reverse.

### Data evaluation and statistical analysis

All experiments were randomized and performed by investigators blinded toward the treatment groups (computer-based randomization). In order to determine the required sample size, the a priori power analysis using G ∗ Power^35^ was performed with the main variable, the primary endpoint, lesion volume data from previously published studies^5,36^. Therefore, based upon the data of these studies the present a priori power analysis was performed to determine an effect size of d 1.75, with an actual standard statistical power (1 − *β*) of 0.95, and a significance level (*α*) of 0.05 and a sample size per group of *n* = 7. In order to have a sufficient power, we decided to have larger sample sizes (*n* = 8 – 12, per group). However, due to shortage of AT2 knockout mice, in the 5 days groups, only sample sizes of 6 were possible. Therefore, these results must be carefully considered. Statistical analysis was performed using the GraphPad Prism 8 Statistical Software (GraphPad Software Inc., La Jolla, CA, USA). Data distribution was tested by Shapiro-Wilks test. The comparisons of parametric and non-parametric data between two independent groups were done using the Welch-*t* test and the Wilcoxon rank sum test, respectively. For the statistical analysis of mNSS we performed ANOVA on ranks with the Kruskal–Wallis test, corrected for multiple comparisons using the Dunn’s test. In this multi-arm parallel group randomized trial, for comparison of multiple independent groups, if the Shapiro–Wilk normality test was passed, one-way analysis of variance (one- way ANOVA) with post-hoc Holm-Šidák comparisons test (comparisons between all groups) was employed. To evaluate group differences in repeated measurements from the same animals (body weight, systolic blood pressure), repeated measures (RM) two-way ANOVA (two-factor repetition) was applied (factors: treatment and time), followed by Šidák’s multiple comparisons test. Whenever there were missing values in the repeated measures dataset and a two-way ANOVA was not possible, repeated measures data were analyzed with the mixed effect model using the restricted maximum likelihood (REML) method with Holm Šidák’s multiple comparison test. Values of *p* < 0.05 were considered significant. Data are presented as the mean and standard deviation (mean ± SD).

### Ethics approval

The studies were performed with the approval of the Animal Care and Ethics Committee of Rhineland-Palatinate, Germany in accordance with the institutional guidelines of the Johannes Gutenberg University, Mainz (protocol numbers: 23177-07/G07-1-021 and 23,177-07/G13-1-046).

### Consent for publication

All authors read and approved the final manuscript.

## Results

### Physiological parameters

Perioperative arterial blood gases were determined 15 min after CCI under general anesthesia in mice (*n* = 6, respectively) with blood gas analyzer ABL800 Basic (Radiometer Medical ApS, Brønshøj, Denmark) in a parallel investigation, which has been published in 2012 by Timaru-Kast et al.^33^: pH: 7.32 ± 0.04, p_a_O_2_: 272 ± 21 mmHg, p_a_CO_2_: 48 ± 4 mmHg. The data shows that in our standardized anesthesia and operation setting, values are stable and within normal physiological limits^33^. In the present study in all groups body temperatures during surgery were within physiological levels.

### Blood pressure was decreased in high dose C21-treated mice 2 h after CCI

**Figure 1:**
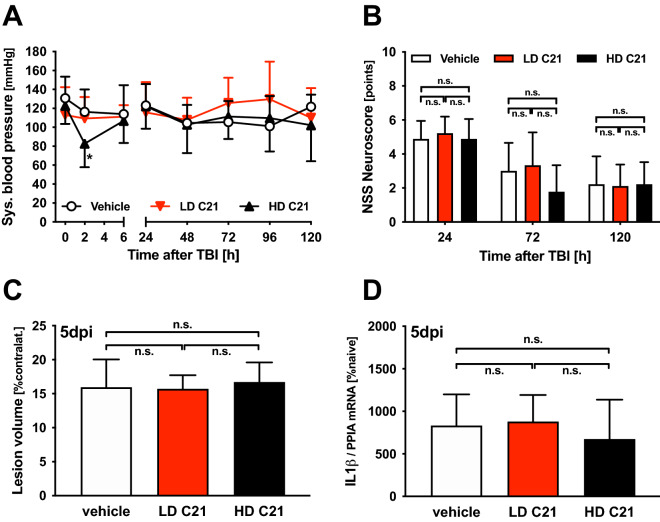
Effect of AT2 activation with C21 on blood pressure, neurological outcome, lesion volume and cerebral inflammation: Vehicle-treated WT mice (*n* = 9; white) were compared to WT mice treated with low (0.03 mg/kg; LD C21; *n* = 9; red), or high dose (0.1 mg/kg; HD C21; *n* = 9; black) of C21; the data are expressed as mean ± SD; n.s. = not significant. (**A**) Peritraumatic systolic blood pressure (time point 0 during anesthesia, postoperative in awake animals): while there were no significant changes of blood pressure in the vehicle and LD groups, in the HD group there was a limited drop of blood pressure 2 h after CCI compared to the vehicle-treated group (*p = 0.035). At later time points, the values were within physiological levels, independent of the treatment. (**B**) NSS: one day (24 h) after TBI in all treatment groups, there was a significant increase in the neurological impairment followed by a significant improvement the following days in all groups. Treatment with C21 in both dosages did not affect neurologic outcome compared to the vehicle. (**C**) The cerebral lesion volume 5 days after TBI (5 days post-injury, dpi; to rule out effects of edema, data are expressed as % contralateral hemisphere) was elevated compared to the primary lesion (*p* < 0.05; see results section); however, there was no difference between treatment groups. (**D**) Five days after TBI (5 dpi), the perilesional gene expression of the cytokine IL1β was not affected by AT2 activation with C21 in both dosages compared to the vehicle treatment.

The systolic blood pressure was measured intraoperatively (time point 0), during anesthesia, and postoperatively in awake animals, 2, 6, 24, 48, 72, 96, and 120 h after TBI. While there were no significant changes in the blood pressure in the LD groups, the HD group had significantly lower blood pressure at 2 h after CCI (82 ± 24 mmHg) compared to the vehicle-treated group (116 ± 24 mmHg; *p* = 0.0353). However, there was no treatment x time interaction for blood pressure and no other differences between the treatment groups were found. The mean systolic blood pressure values were 115 ± 24 mmHg in the vehicle group, 116 ± 28 mmHg in LD, and 107 ± 27 mmHg in the HD group (Fig. 1A).

### Neurological outcome and body weight after TBI were not affected by C21 treatment

There was a significant increase in the neurological impairment according to NSS at day 1 post-TBI in all groups, followed by a significant improvement in the following days. However, the neurologic outcomes in the LD and HD groups were not significantly different from those in the vehicle-treated mice (Fig. 1B). Furthermore, the body weight development was similar in all groups. After a decrease at day 1 after CCI body weight increased significantly throughout the observation period (19.7 ± 1.8, 19.7 ± 1.5 and 20.1 ± 1.8 g vs. 20.1 ± 0.7*, 20.7 ± 1.7* and 21.0 ± 1.3* g for vehicle, low and high dose C21 treated mice at day 1 versus day 5, *p < 0.05; Table 3). Increase of body weight was earlier significant compared to day 1 in C21 treated mice (at day 4). However, both dosages C21 did not affect body weight compared to vehicle treated mice at any time point (Table 3).

**Table 3:** Body weight in vehicle and C21 treated mice.

Group	Before CCI	Day 1	Day 2	Day 3	Day 4	Day 5
Vehicle	19.8 ± 1.9	19.7 ± 1.8	19.8 ± 1.7	20.0 ± 1.7	20.3 ± 1.6	20.1 ± 0.7*
C21 LD	20.2 ± 1.7	19.7 ± 1.5	20.2 ± 1.5	20.4 ± 1.5	20.6 ± 1.5*	20.7 ± 1.7*
C21 HD	20.2 ± 1.9	20.1 ± 1.8	20.3 ± 2.1	20.6 ± 1.7	20.9 ± 1.6*	21.0 ± 1.3*

Bodyweight of vehicle, low (LD C21) and high dose C21 (HD C21) treated mice before CCI and day 1 to 5 after CCI in g (*p < 0.05 vs. Day 1).

In a confirmatory investigation in another set of mice with the same treatment regime, but with reduced CCI settings and consecutively 45% smaller lesion volumes, NSS at days 1, 3 and 5 after TBI was without difference between C21-treated groups and vehicle-treated mice (NSS: vehicle: 4 ± 3, 3 ± 2, 1 ± 2; LD: 4 ± 2, 4 ± 2, 2 ± 2; HD: 3 ± 2, 2 ± 2, 1 ± 1 points, respectively).

### Lesion volume was not affected by C21 treatment

The primary lesion volume was determined 15 min after CCI (9.2% ± 1.2% contralateral hemisphere). Five days after CCI, the lesion volume was significantly enhanced compared to the primary lesion volume (*p* < 0.05); however, there was no difference between groups (15.9% ± 4.1%, 15.7% ± 2.0%, and 16.7% ± 2.9% contralateral hemisphere volume for vehicle, LD, and HD, respectively; Fig. 1C). As was found for the initial study, the confirmatory study with altered CCI device settings also demonstrated no significant between-group changes (9.9% ± 1.9%, 9.4% ± 1.9%, and 8.9% ± 1.9% contralateral hemisphere volume for vehicle, LD, and HD, respectively).

### Gene expressions of neurotrophin receptors were upregulated, while expressions of neurotrophins and cytokines were unaffected by C21 treatment

**Figure 2:**
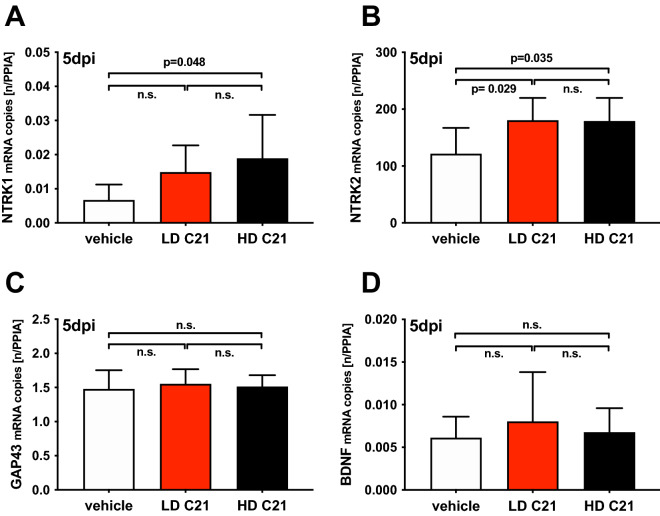
Effect of AT2 activation with C21 on neurotrophin receptor and effector expression: Vehicle-treated WT mice (*n* = 9; white) were compared to WT mice treated with low (0.03 mg/kg; LD C21; *n* = 9; red), or high dose (0.1 mg/kg; HD C21; *n* = 9; black) of C21 at 5 days post-injury (5dpi); the data are expressed as mean ± SD; n.s. = not significant. (**A**)**, (B**) There were dose-dependent elevated gene expressions of the neurotrophin receptors NTRK1 in HD C21 and of NTRK2 in LD and HD C21 compared to those in the vehicle-treated mice (*p* < 0.05). (**C**)**,** (**D**) The gene expressions of the neurotrophins GAP43 and BDNF were not regulated differently between the vehicle- and C21-treated mice.

Five days after TBI, the perilesional gene expression of the cytokine IL1β, neurotrophin receptors NTRK1 and NTRK2, and neurotrophins GAP43 and BDNF, as assessed by qPCR, were elevated compared to naïve mice (*p* < 0.05). IL1β was not affected by C21 treatment (Fig. 1D). A dose-dependent upregulation of the neurotrophin receptors NTRK1 (in the HD group only; *p* = 0.048) and NTRK2 (in both LD and HD groups; *p* = 0.029 and *p* = 0.035; Fig. 2A, B) was found. However, gene expression of the neurotrophins GAP43 and BDNF was not significantly different between the vehicle and LD- or HD-group mice. (Fig. 2C, D).

### AT2 knockdown did not create significant differences from WT mice

**Figure 3:**
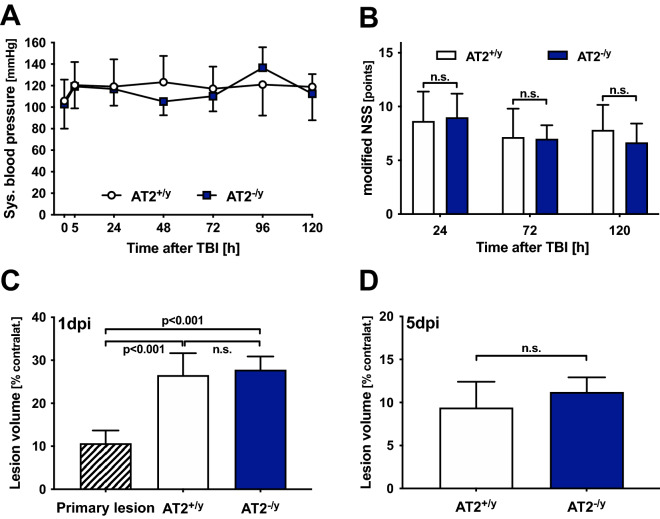
AT2 deficiency does not affect the blood pressure, neurological outcome and the lesion volume: AT2-deficient mice (AT2^−/y^; 24 h: *n* = 12; 5 days: *n* = 6; blue) were compared to age-matched WT mice (AT2^+/y^; 24 h: *n* = 12; 5 days: *n* = 6; white). The values are presented in mean ± SD; n.s. = not significant. (**A**) Peritraumatic systolic blood pressure: time point 0 represents the intraoperative measurement immediately after CCI under general anesthesia, and the following postoperative time points measurements were performed in awake animals. The values were within the physiological norms and did not differ between the AT2^−/y^ and AT2^+/y^ animals. (**B**) The mNSS after CCI was not significantly different between the AT2^−/y^ and AT2^+/y^ animals. (**C**)**,** (**D**) Cerebral lesion volume (to rule out effects of edema, data are expressed as % contralateral hemisphere): primary lesion was assessed 15 min after TBI, without difference between AT2^+/y^ and AT2^−/y^ animals. **C:** The marked secondary increase of lesion volume 24 after TBI (1 day post-injury, 1dpi; *p* < 0.001) was not different in AT2^+/y^ mice compared to AT2^−/y^ mice. (**D**): Five days after TBI (5dpi), there was no difference in the lesion volumes between the AT2^+/y^ and AT2^−/y^ mice.

The systolic blood pressure was within physiological levels and not significantly different between the AT2-knockdown and WT mice (Fig. 3A).

In study II, the neurological status was determined with mNSS. The mNSS scores were not significantly different between the AT2^+/y^ and AT2^−/y^ groups; however, there was a significant increase of neurological impairment 24 h after CCI, followed by a gradual improvement in the following days in all groups (Fig. 3B). Age matched AT2 deficient and wild type litter mate mice were used. Their body weight decreased after CCI continuously from before TBI during 5 days after TBI in both groups. However, AT2 deficiency had no impact on body weight loss after brain trauma (37.8 ± 6.7 vs. 41.2 ± 5.7, 36.3 ± 7.2* vs. 39.3 ± 5.6*, 35.1 ± 6.5*^#^ vs. 37.8 ± 5.5*^#^ and 34.7 ± 7.0*^#^ vs. 37.5 ± 5.1*^#^ g in AT2^+/y^ vs. AT2^-/y^ at time points before CCI, 1 day, 3 days and 5 days after CCI, respectively; *p < 0.05 vs. before TBI, ^#^*p* < 0.05 vs. day 1 after CCI). The primary injury volumes of AT2^−/y^ (9.0% ± 1.5% contralateral hemisphere) and AT2^+/y^ (12.2 ± 3.2% contralateral hemisphere) were not significantly different (15 min after CCI). Thus, the primary injury volumes of both groups are presented together. There was a significant increase in the lesion volume 24 h after TBI due to secondary damage compared to the primary lesion volume measured 15 min after TBI (primary injury volume, 10.7% ± 3.0% contralateral hemisphere; secondary injury volume, 26.6% ± 5.1% contralateral hemisphere (WT); secondary injury volume, 27.8% ± 3.1% contralateral hemisphere (AT2^−/y^)). Due to scar tissue formation, compared to 24 h post-CCI, the lesion volumes at day 5 were reduced in AT2^+/y^ and AT2^−/y^ animals (10.4% ± 2.1% and 11.2% ± 1.7% contralateral hemisphere for AT2^+/y^ and AT2^−/y^, respectively). Neither post-CCI time point demonstrated between-group differences (Fig. 3C, D).

**Figure 4:**
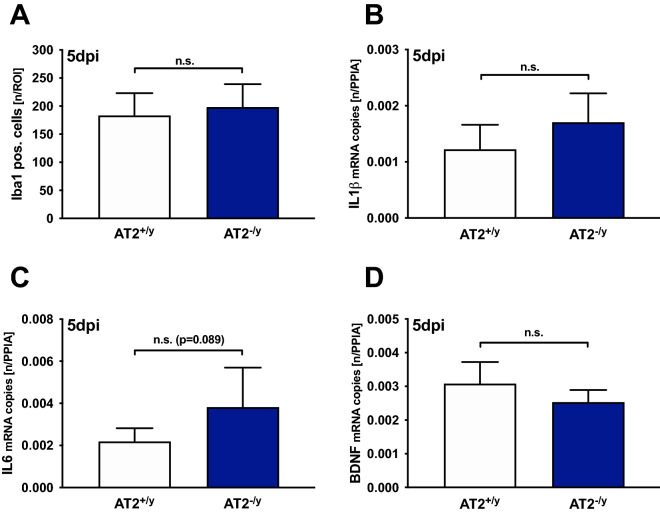
AT2 deficiency does not affect the neuroinflammation and neurotrophins: AT2-deficient mice (AT2^−/y^; *n* = 6; blue) were compared to age-matched WT mice (AT2^+/y^; *n* = 6; white) 5 days after TBI (5 dpi). Values are presented in mean ± SD; n.s. = not significant. (**A**) The number of Iba-1-immunolabelled activated microglia/macrophages 5 days after CCI in the perilesional region of interest (0.52 × 0.65 mm^2^) was not different between the AT2^+/y^ and AT2^−/y^ groups. (**B**)**,** (**C**)**,** (**D**) The perilesional gene expressions of the cytokines IL1β and IL6 and of the neurotrophin BDNF, determined by qPCR, were not different between the AT2^+/y^ and AT2^−/y^ mice 5 days after TBI (5 dpi).

We assessed the number of perilesional microglia/macrophages as a proxy of microglial activation using anti-Iba-1 immunohistochemistry 5 days after CCI in a perilesional ROI (0.52 × 0.65 mm^2^). The rationale for measuring IBA-1 in an area adjacent to the lesion rather than within the lesioned area was, that inside the lesion, where the tissue is essentially destroyed, microglia/macrophages are almost absent. In the perilesional area, however, there is a robust activation of microglia/macrophages. The numbers of perilesional Iba-1-immunolabelled microglia/macrophages were substantially increased as compared to corresponding sites in the non-injured contralateral hemisphere. However, in the perilesional and in the corresponding contralateral region they were similar between the AT2^+/y^ and AT2^−/y^ groups (perilesional: 183 ± 40** and 198 ± 41** n/ROI, contralateral: 78 ± 7 and 69 ± 7 n/ROI, **p < 0.001 compared to contralateral corresponding region, for AT2^+/y^ and AT2^−/y^, respectively; Fig. 4A). In addition, the gene expressions IL1β and IL6 (Fig. 4B,C) were not significantly different between AT2^+/y^ and AT2^−/y^ 5 days after TBI. No other measured genes (TGFβ, GAP43, and BDNF; Fig. 4D) demonstrated significant differences between the knockdown and WT groups. Finally, no differences in NTRK1/2 expressions between groups were found.

## Discussion

Several studies demonstrated sustained protective effects by blockade of AT1 after TBI^5,37^, and a neuroprotective role for AT2 in ischemic stroke^17,25,38^, spinal cord injury^10^ and nerve regeneration ^15^. In spite of pathophysiological similarities between brain ischemia and TBI^39^, the therapeutic potential of the non-peptide selective AT2 agonist C21 after TBI has only been the subject of few studies to date. However, despite the seeming potential for success, in our murine CCI model of TBI, we found that direct AT2 stimulation by repeated application of C21 at high and low dosages did not produce any significant neuroprotection up to 5 days after TBI. Therefore, as proof of concept we investigated the effect of AT2 knockdown on our model system, and again found no differences. The present data surprisingly contradicted our foregoing assumption that AT2 stimulation exerts neuroprotection after TBI. Indeed, there are striking differences between our protocols and TBI models that showed protection by AT2 stimulation. Each of the possible TBI models with inherent advantages and disadvantages, need to be thoroughly considered according to the hypothesis and design of the study when selecting the rodent TBI model^40^. In a closed-head injury study, Umschweif and colleagues demonstrated reduction of lesion size and improvement in functional recovery by treatment with the peptide AT2 agonist CGP42112A^24^. The closed-head injury model is designed to replicate the biomechanics and pathobiology of concussive brain injury^41,42^. The impact of the standardized weight-drop device with dynamic pressure to the closed skull and acceleration and deceleration trauma results predominantly in diffuse brain injury^41,43–46^. On the other hand, CCI is a very common model that simulates focal TBI with cortical contusion, brain edema, elevated intracranial pressure, and decreased cerebral blood flow^41,47^. The murine CCI model offers a very accurate control of the mechanical components of trauma. By exact adjustment of the impact velocity and depth, it is possible to generate a defined, focal cortical lesion with high reproducibility^41,48^. To rule out the possibility of a too large traumatic brain damage where putative protective effects of AT2 activation may be diminished, we repeated the present study in a confirmatory study with reduced brain penetration of the impactor tip and, apart from that, the same CCI settings and treatment regime. This change of CCI settings lead to a reduced brain damage. However, AT2 activation with C21 in high and low dosages had still no effect on lesion size, compared to vehicle treated mice. Despite equal CCI settings, lesion volumes 5 days after TBI were smaller than 24 h after TBI, due to reorganization of astrocytes, phagocytosis of necrotic and apoptotic cells and debris^49,50^. Besides expansion of lesion volume, CCI lead to a robust activation of microglia, with increased number of perilesional microglia as compared to corresponding non-injured contralateral regions after CCI^51,52^, and compared to corresponding brain regions in sham mice^53^. Besides different TBI models, in contrast to Umschweif et al., the focus of the present study was the early time phase after TBI, and motor impairment and behavior rather than cognitive outcomes by using the NSS^29^.

One major difference to other related studies was our use of C21. The reason we chose the non-peptide AT2 agonist C21 was, that the peptide alternative CGP42112A has unfavorable pharmacokinetic properties such as very rapid degradation. It has to be applied continuously ^24^, and therefore in the past it never has been considered to be developed for clinical use^54^. To model a clinical treatment as possible therapeutic opportunity of AT2 activation after TBI was one aim of the present study^14,54–56^. Therefore, we used C21, the non-peptide AT2 agonist with a more favorable pharmacokinetic profile, as repetitive post-TBI treatment (starting 30 min after CCI) with the potential to be developed in further studies to a possible novel pharmacological concept^10^. The AT2-specificity of C21 has been proven in several studies^10,25,55,57^. The decision to choose the two doses of C21 was made, based upon dosages that have shown to be protective in ischemic brain damage, in spinal cord and cardiac injury. The dosage of 0.03 mg/kg/day C21 has been demonstrated to be cardioprotective and to decrease systemic inflammation after left coronary artery occlusion^58^, higher doses (0.3 mg/kg/day) C21 did not increase cardio-protection^58^. In a recent rodent brain ischemia study (1.5–3 h after MCAO), it was shown that i.p. treatment with C21 (0.03 mg/kg) at reperfusion downregulated apoptotic and oxidative markers, increased neurotrophin activity, reduced inflammation and infarct size and improved behavioral outcome at 24 h without affecting the blood pressure^59^. In the present study, our dosage of C21 was chosen based on the murine study of Schwengel et al.^25^, wherein the effect of post-stroke C21 treatment after MCAO was assessed in AT2^−/y^ and AT2^+/y^ mice. The study demonstrated that 0.03 mg/kg/day C21 significantly improved the survival and neurologic outcomes after MCAO compared to the vehicle treatment, although it had no impact on infarct size 96 h post-stroke in AT2^+/y^ mice^25^. Expression of BDNF and its receptor NTRK2 and GAP43 were significantly increased in the peri-infarct cortex of C21-treated, compared to vehicle-treated WT mice^25^. Therefore, based upon these data, we decided to use two doses (0.03 and 0.1 mg/kg/day) to reduce the possibility of under-dosage and investigate the dose–response relationship.

Passage through the blood brain barrier (BBB) and availability of C21 in the cerebral tissue is substantial for the effectivity of C21. Indeed, the lack of measuring the effect site concentration of C21 in the brain tissue after TBI, is a limitation of the present study. In naïve rodents, after i.p.-injection, recent concentration analyses showed only minimal passage of C21 through the intact BBB^60,61^. After ischemic stroke, however, 30 min following i.p.-injection of 0.03 mg/kg, levels of C21 within the brain cortex and striatum were significant^61^. Several studies have shown a peak in permeability in the acute and subacute phase followed by a gradual reduction of BBB permeability after ischemic stroke^62^. Rodent models of TBI have demonstrated a biphasic increase in the BBB permeability to albumin and other high- molecular-weight proteins peaking at 4–6 h and 2–3 days after injury^63,64^. In our present model, there is a distinct BBB disruption with high permeability peaking at 6–24 h after CCI, as it has been shown earlier by Luh and coworkers^32^. In human TBI, BBB disturbances can be detected from several days up to years after the trauma^65^. On the basis of these findings we postulate, that due to post-traumatic increased permeability of the disturbed BBB and repetitive treatment, in the present study there was an adequately high effect site concentration of C21 for receptor stimulation of AT2 in the injured brain tissue^55^.

In a recent preliminary murine CCI-study with C21 (0.03 mg/kg, i.p.; compared to saline) treatment at 1 h and 3 h post-TBI^66^, NSS was improved and inflammatory markers, like IL1β reduced at 24 h post-TBI in the pericontusional areas. However, lesion volume was not measured in this preliminary study. In a recent study we demonstrated that IL1β expression was increased in the first 6 h after TBI ^5^. However, C21 did not affect IL1β expression 5 days after TBI herein.

Moreover, in the present study, the primary endpoint lesion volume was not affected by treatment with C21. This is in accordance with recent studies with spinal cord injury^10^ and brain ischemia. Schwengel and coworkers noted reduced mortality and neurological impairment by post-stroke treatment with C21 without effect on the infarct size^25^. Therefore, AT2 activation with C21 may have no effect on the secondary brain damage expansion. Lee and colleagues showed in a cerebral ischemia model (MCAO) in mice the neuroprotective effects of the peptide AT2 agonist CGP42112 with reduced infarct volumes, while treatment with the non-peptide C21 failed to show protective properties and had no effect on the neuronal survival^67^ in an in vitro glucose deprivation model. The authors found that unlike the peptide AT2 agonist CGP42112, there was no protective effect by a similar range of concentrations of C21^68^. However, the focus of the present study was to investigate a possible in vivo effect of AT2 activation by C21 after TBI in mice with dosages that have been shown to be protective in earlier rodent models^25,43,59^. We do not believe under-dosage was a problem in the present study despite the relative lack of previous data to draw on. Possible proof of activity of C21 was found in the elevated expressions of the neurotrophin receptors NTRK1 and NTRK2 and in blood pressure reduction 2 h after TBI in the HD compared to vehicle group. However, blood pressure was not altered at most of the time points. This is in line with several studies that showed no or only slight changes in blood pressure with C21 therapy^69,70^.

Thus, the different TBI model (closed head TBI versus CCI) and the different AT2 agonists (peptide vs. C21) could have led to the different results. Several issues arose by our negative results. Limitations by the substance or by the application protocol may have contributed to the failure to detect a positive effect on the outcome measures in the model. Pre-treatment with C21 may have had different results. Therefore, after performing the experiments with C21, to rule out underdosage or wrong timing of application, we decided to analyze AT2 knockout mice without additional treatment, as proof-of-concept. We investigated the effect of AT2 deficiency 24 h and 5 days after CCI, however, without differences in brain damage, neuroinflammation and neurology.

A possible reason for our negative results, especially those in the AT2-knockdown mice, is the possibility of a non-protective, proinflammatory role of AT2. Ruiz-Ortega and coworkers demonstrated that AngII increased the proinflammatory Nuclear factor-kappaB (NF-κB) activation via AT1 and AT2 in rat smooth muscle cells^71,72^. Although NF-κB-mediated transcription occurred mainly through AT1, AT2 activation enhanced NF-κB-DNA-binding and NF-κB-mediated transcription of cytokines^71^. Furthermore, in AT1-knockdown mice, an increase of NF-κB could be demonstrated by AngII stimulation. Blockade of AT2, however, led to a drop of NF-κB in these mice^71–74^. Additionally, Wolf investigated the AngII receptor subtypes involved in NF-κB activation, demonstrating in various cell lines that Ang II induces NF-κB activation through AT2 receptor stimulation^75^. Sabuhi tested the effects of the AT2 activation with CGP42112A on inflammation and oxidative stress in obese Zucker rats and their lean counterparts. While AT2 activation reduced inflammatory and oxidative stress markers in obese rats, it increased such markers in lean rats. These results suggest that AT2 receptors may have pro- or anti-inflammatory effects according to weight or weight-related factors^76^. Furthermore, previous studies indicate that neuroprotection by AT2 activation is predominantly mediated by concomitant AT1 antagonism^17,67^. However, aim of the present study was to investigate the role of AT2, examine the effect of AT2 activation by C21 in the acute phase after TBI. However, the question if AT2 activation is neuroprotective with concomitant AT1 inhibition in TBI pathophysiology has to be addressed in future studies.

In summary, in contrast to studies of AT1 inhibition ^5^, a selective AT2 activation with C21 did not reduce brain damage in the CCI model. Therefore, the present results suggest that neuroprotective properties of AT1 inhibition in the early phase after TBI are not due to enhanced activation of AT2 by AngII. However, the neuroprotective properties of AT2 that contribute to reparative processes may be present in later phases after TBI.

## Conclusion

Several recent studies have shown that inhibition of AT1 improved neurological outcome and reduced secondary brain damage after experimental TBI. There is growing evidence that AT2-mediated protective mechanisms may contribute to the protective potency of AT1 inhibition. In the present study, the effect of AT2 activation on neurologic outcome, cerebral inflammation, and lesion volume was assessed for the first time after CCI. The present data could not confirm a protective role of AT2 activation, nor did AT2 deficiency affect brain damage within 5 days after TBI. This suggests that AT2 may play a minor role in the early phase after TBI. The present results further suggest that neuroprotective properties of AT1 inhibition are not due to enhanced activation of AT2 by AngII within the first five days, in the early phase after TBI. Further investigations are required to assess later effects of AT2 activation by C21 after TBI.

## Data Availability

The datasets generated during and/or analyzed during the current study are available from the corresponding author on reasonable request.
